# Patients’ willingness to participate in clinical trials and their views on aspects of cancer research: results of a prospective patient survey

**DOI:** 10.1186/s13063-015-1105-3

**Published:** 2016-01-09

**Authors:** Sing Yu Moorcraft, Cheryl Marriott, Clare Peckitt, David Cunningham, Ian Chau, Naureen Starling, David Watkins, Sheela Rao

**Affiliations:** The Royal Marsden NHS Foundation Trust, London, UK; The Royal Marsden NHS Foundation Trust, Sutton, UK

**Keywords:** Patient attitudes, Biopsies, Cancer research, Clinical trial recruitment, Consent, Patient feedback, Patient information, Trial ineligibility

## Abstract

**Background:**

Recruitment to clinical trials can be challenging and slower than anticipated. This prospective patient survey aimed to investigate the proportion of patients approached about a trial who agree to participate, their motivations for trial participation and their views on aspects of cancer research.

**Methods:**

Patients who had been approached about participation in any clinical trials in the Gastrointestinal and Lymphoma Unit at the Royal Marsden were invited to complete a questionnaire. The statistical analysis is mainly descriptive, with percentages being reported. Univariate logistic regression analysis was used to determine any associations between patient characteristics and patient responses.

**Results:**

From August 2013–July 2014, 276 patients received 298 clinical trial patient information sheets and were asked to complete the questionnaire. The majority of patients (263 patients, 88 %) consented to a clinical trial and 249 of the 263 patients (95 %) completed the questionnaire. Multiple factors influenced decisions to participate in clinical trials, with patients stating that the most important reasons were that the trial offered the best treatment available and that the trial results could benefit others. Of the 249 questionnaire respondents, 78 % would donate their tissue for genetic research, 75 % would consider participating in studies requiring a research biopsy and 75 % felt that patients should be informed of trial results. Patients treated with palliative intent and those who had received multiple lines of treatment were more willing to consider research biopsies. Of the patients approached about a clinical trial of an investigational medicinal product, 48–50 % would have liked more information on the study drugs/procedures.

**Conclusion:**

The majority of patients approached about a clinical trial consented to one or more trials. Patients’ motivations for trial participation included potential personal benefit and altruistic reasons. A high proportion of patients were willing to donate tissue for research and to consider trials involving repeat biopsies. The majority of patients feel that participants should be informed of trial results and there is a group of patients who would like more detailed trial information.

**Electronic supplementary material:**

The online version of this article (doi:10.1186/s13063-015-1105-3) contains supplementary material, which is available to authorized users.

## Background

Recruitment to clinical trials can be challenging, leading to 1 in 10 cancer trials registered on ClinicalTrials.gov between 2005 and 2011 being closed prematurely due to poor accrual and other trials taking longer than anticipated to complete recruitment [1–3]. This is a major problem for oncologists, not only from a scientific perspective, but also due to the implications of failing to meet recruitment targets. For example, in the United Kingdom (UK), clinical trial performance metrics include meeting certain government targets, such as recruiting the first study patient within 70 days of receiving a valid research application [4]. Although the UK has one of the highest clinical trial participation rates in the world, with more than 1 in 5 adult cancer patients participating in clinical trials [5], the proportion of patients with whom cancer research is discussed ranges from 10–61 % depending on the hospital trust [6].

Patients’ decisions regarding trial participation may be influenced by the information they receive. Trials are becoming larger and increasingly complex, incorporating translational research (including research biopsies), adaptive designs and biomarker selection/stratification [7, 8]. However, the level of scientific and health literacy in the general population remains poor, with a third of older adults in England having difficulty in reading and understanding basic health-related information [9] and previous studies have shown that patients can have misconceptions about aspects of research, such as the risk of side effects, the trial’s aims and the likelihood of personal benefit [10–12]. Therefore, there is concern that clinical trial patient information sheets (PIS) may not be fit-for-purpose. In addition, there are concerns that mandatory research biopsies may deter patients from participating in trials and, therefore, adversely affect trial recruitment [13, 14].

A better understanding of patients’ motivations for participating in cancer research and their opinions of trial information and the consent process may lead to changes that facilitate trial recruitment and improve patient satisfaction with the recruitment process. We therefore conducted a prospective patient survey of patients treated at the Royal Marsden (RM) for gastrointestinal (GI) cancers or lymphoma in order to investigate patients’ willingness to participate in clinical trials and their views on aspects of cancer research.

## Methods

The patient survey was approved by the Royal Marsden Committee for Clinical Research and patients verbally consented to participate in the survey. This survey was designed as a ‘service evaluation’, to evaluate patients’ experiences of clinical trials at our institution and thereby investigate if aspects of this process could be improved. Ethical review by an external review board was, therefore, not required. The primary endpoint was the proportion of patients approached about a clinical trial who agreed to participate in a trial. Secondary endpoints included reasons why patients consented to/declined trials, the proportion of patients who were happy to be approached about participating in research, the proportion of patients who would consider trials involving genetic research or research biopsies and patients’ views of the consent process, the trial information provided and feedback of study results to participants.

### Study subjects

All patients who had been approached about a trial between August 2013 and July 2014 in the GI and Lymphoma unit at the RM (a specialist cancer centre) were eligible to participate in this patient survey. Clinical Trials of an Investigational Medicinal Product (CTIMP) and non-CTIMP trials were included, as well as pre-screening studies. The CTIMP trials included trials investigating targeted therapies, immunotherapies, chemotherapy, the optimal duration of chemotherapy and the scheduling of chemotherapy and surgery (e.g. peri-operative chemotherapy versus post-operative chemotherapy).

Patients were invited to participate in the survey by the unit research nurses or doctors when they notified staff about their decision regarding participation in a trial. They were also informed that the survey aimed to improve the experiences of patients with regards to clinical trials and that participation in the survey was voluntary with no obligation to take part.

### Questionnaires

Two paper questionnaires were developed based on a literature review and the authors’ experiences of trial recruitment. The questionnaires were comprised of Likert, multiple choice and free-text questions. Patients who consented to a trial were given the 25-question Questionnaire A (Additional file 1), which included questions on their reasons for deciding to participate in the trial. Patients who declined a trial received the 21-question Questionnaire B (Additional file 2), which included questions regarding their reasons for declining the trial. Patients who consented/declined more than one trial were asked to complete one questionnaire per trial. The questionnaires could be completed in clinic or taken home and returned at the patients’ next clinic appointment.

Each patient was allocated a unique survey ID number, which was written on their completed questionnaires and on a demographic sheet completed by the research nurse looking after the patient. The researchers analysing the survey responses were not able to identify the patients from their survey ID numbers. The questionnaire was, therefore, anonymous to the researchers analysing the survey results, unless the patient chose to complete an optional section of the questionnaire and provided their details so that they could be contacted about future surveys.

SYM and CM compared patients’ free-text answers to the question: ‘what type of cancer do you have?’, with the data collected on the demographic sheet and scored the answers as being correct if the patient had clearly identified the site of their primary tumour.

### Collection of demographic and trial-related information

Demographic information was collected by research nurses from patients’ electronic medical records. Social class was characterised according to the National Readership Survey (NRS) classification [15] by SYM and CM according to the patients’ recorded occupation. The percentage of households in poverty in the patients’ postcode area was determined using data available from the Office for National Statistics, UK [16], and used as a marker of social deprivation. Data on clinical trial characteristics, PIS characteristics, whether the patient agreed to complete the questionnaire and subsequent trial registration (including reason for trial ineligibility) was also recorded.

### Statistical analysis

The majority of the statistical analysis is descriptive, with percentages being reported. The statistical analysis was performed using Stata v13.1 (StataCorp, College Station, TX, USA). Univariate logistic regression analysis was used to determine any associations between patient characteristics and patient responses and between trial characteristics and PIS features. Patient characteristics included in the univariate analysis were age, gender, performance status (PS), number of previous lines of treatment, previous trial participation, aim of treatment (curative versus palliative) and type of trial.

## Results

### Clinical trial portfolio and patient recruitment

**Fig. 1:**
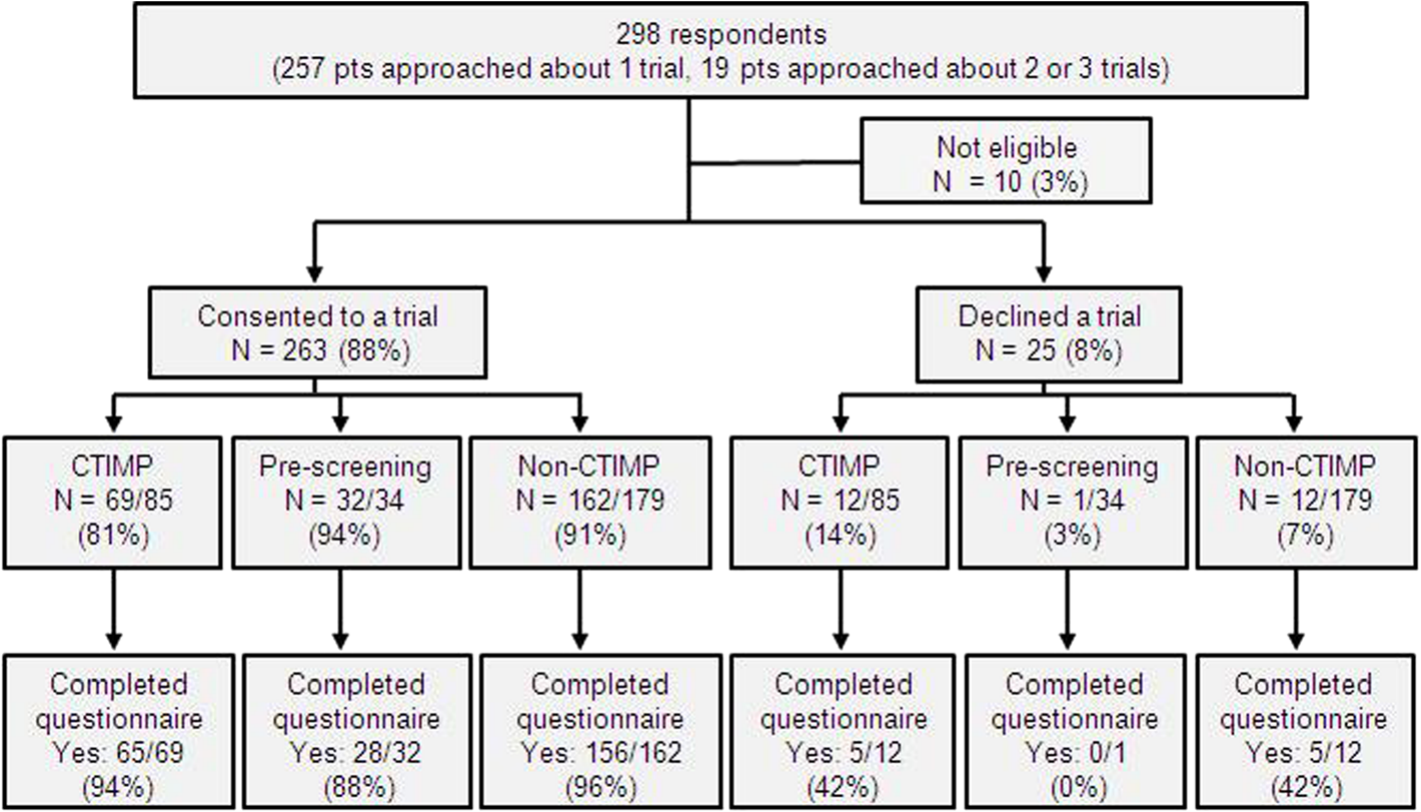
Recruitment to clinical trials and the patient survey

Between August 2013 and July 2014, 36 trials recruited one or more patients (see Table 1). Two hundred and seventy-six patients received 298 PIS for a clinical trial (271 GI, 27 lymphoma), with 257 patients receiving 1 PIS, 16 patients receiving 2 PIS and 3 patients receiving 3 PIS. Patient demographics are shown in Table 2. Two hundred and sixty-three patients (88 %) consented to a trial and 249 (95 %) of these 263 patients completed the questionnaire (see Fig. 1). Ten patients were ineligible for the trial at the time they returned for consent (e.g. due to clinical deterioration) and were, therefore, ineligible for the patient survey.

**Table 1:** Characteristics of trials in the Gastrointestinal and Lymphoma Unit (*n* = 36)

Trial characteristic	Number of trials (%)
	(*n* = 36)
Study type	
CTIMP	24 (67 %)
Non-CTIMP	9 (25 %)
Pre-screening	3 (8 %)
Sponsor	
Royal Marsden	9 (25 %)
Other academic institution	11 (31 %)
Pharmaceutical company	16 (44 %)
Phase	
I	2 (6 %)
II	10 (28 %)
II/III	2 (6 %)
III	13 (36 %)
Not applicable	9 (25 %)
Trial setting	
Neoadjuvant	3 (8 %)
Adjuvant	3 (8 %)
Advanced	16 (44 %)
Any	5 (14 %)
Lymphoma^a^	9 (25 %)
Randomised trial	
Yes	20 (56 %)
No	16 (44 %)
Molecular screening	
Yes	10 (28 %)
No	26 (72 %)
Number of PIS (e.g. separate pharmacodynamics or imaging sub-studies)	
1	20 (56 %)
2	11 (31 %)
3	4 (11 %)
5	1 (3 %)

Key: CTIMP = Clinical Trial of an Investigational Medicinal Product, pre-screening = molecular pre-screening to determine potential eligibility for a specific CTIMP study, PIS = patient information sheet

^a^Lymphoma trials were considered separately as the intent of lymphoma treatment is to induce remission and, therefore, the treatment paradigms differ from that of gastrointestinal malignancies

**Table 2:** Patient demographics

Characteristic	*N* (%)
	(*n* = 276)
Gender	
Male	188 (68 %)
Female	88 (32 %)
Age	
Median (range)	64 years (19–85)
Native English speaker	252 (91 %)
Ethnicity	
White	238 (86 %)
Asian	21 (8 %)
Black	8 (3 %)
Other	9 (3 %)
Marital status	
Married/partner	194 (70 %)
Single	35 (13 %)
Separated/divorced	15 (5 %)
Widowed	14 (5 %)
Unknown	18 (7 %)
Social class	
Grade A/B (upper middle/middle class)	56 (20 %)
Grade C1/2 (lower middle/skilled working class)	34 (12 %)
Grade D (working class)	48 (17 %)
Grade E (retired)	109 (40 %)
Grade E (unemployed)	5 (2 %)
Unknown	24 (9 %)
Percentage of households in poverty in the patient’s postcode area	
≥ 27.61 %	20 (7 %)
22.01–27.6 %	43 (16 %)
17.91–22 %	48 (17 %)
14.41–17.9 %	51 (18 %)
≤ 14.4 %	112 (41 %)
Not in England^a^	2 (1 %)
Type of cancer	
Colorectal	117 (42 %)
Oesophagogastric	100 (36 %)
Pancreatic	18 (7 %)
Hepatobiliary	11 (4 %)
Carcinoma of unknown primary/other GI	3 (1 %)
Hodgkin’s lymphoma	10 (4 %)
Non-Hodgkin’s lymphoma	17 (6 %)
Treatment aim	
Curative	96 (35 %)
Palliative	180 (65 %)
Number of previous lines of treatment	
0	133 (48 %)
1	87 (32 %)
2	39 (14 %)
3	13 (5 %)
≥ 4	3 (1 %)
Unknown	1 (1 %)
Performance status	
0	106 (38 %)
1	130 (47 %)
2	20 (7 %)
3	3 (1 %)
Unknown	17 (6 %)

^a^No comparable poverty statistics available for regions outside of England

*GI* gastrointestinal

### Reasons for trial ineligibility

Thirty-eight (14 %) of the 263 patients who consented to a clinical trial were not subsequently registered for the trial, including 36 (36 %) of the 101 patients who consented to CTIMP/pre-screening trials. The main reason for trial ineligibility was that their molecular profile (e.g. human epidermal growth factor receptor 2 (HER2) status) did not meet the study requirements (*n* = 22). Other reasons for patient ineligibility included: no available tissue for molecular testing (*n* = 3), further investigations revealed a change in disease extent: e.g. from localised to metastatic disease (*n* = 3), blood results outside of a specific range (*n* = 3), deterioration in PS (*n* = 2) and non-measurable disease on imaging (*n* = 1).

### Reasons for trial participation

**Fig. 2:**
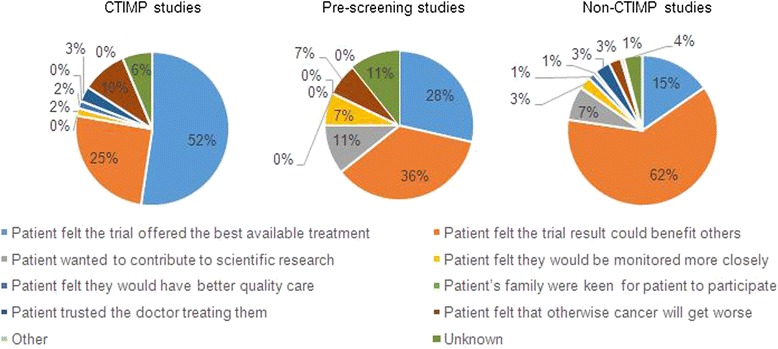
The main reason that motivated patients to participate in a clinical trial. Key: CTIMP = Clinical Trial of an Investigational Medicinal Product, pre-screening = molecular pre-screening to determine potential eligibility for a specific CTIMP study

Multiple factors influenced patients’ decisions to participate in a clinical trial (see Table 3). When patients were asked to indicate their main reason for trial participation, a belief that ‘the trial offered the best treatment available’ or that ‘the trial results could benefit others’ were the most frequent responses (see Fig. 2). A univariate analysis was used to determine any associations between patient characteristics and patients’ main reason for trial participation. Patients were more likely to state that their main reason for participation was ‘the trial offered the best treatment available’, if they were being treated with palliative rather than curative intent (33 % versus 19 %, odds ratio 2.11, 95 % CI 1.09–4.08, *p* = 0.026), had a worse PS (19 % for PS 0, 34 % for PS 1, odds ratio 2.12, 95 % CI 0.97–8.79, *p* = 0.027) or had not previously participated in a clinical trial (32 % (no) versus 8 % (yes) for previously participated, odds ratio 5.14, 95 % CI 1.51–17.5, *p* = 0.009). Patients were more likely to state that their main reason for participation was ‘the trial results could benefit others’ if they were < 65 years compared to ≥ 65 years (64 % versus 39 %, odds ratio 2.77, 95 % CI 1.62–4.74, *p* < 0.001), being treated with curative rather than palliative intent (63 % versus 47 %, odds ratio 1.97, 95 % CI 1.23–3.46, *p* = 0.017) or had previously participated in a clinical trial (72 % (yes) versus 50 % (no) for not participated, odds ratio 2.65, 95 % CI 1.21–5.83, *p* = 0.012). Gender and number of previous lines of treatment did not significantly influence patients’ main reason for trial participation.

**Table 3:** Factors which influenced patients’ decision to participate in a clinical trial

Reason	CTIMP	Pre-screening	Non-CTIMP	All
	*N* (%)	*N* (%)	*N* (%)	(*n* = 241)
	(*n* = 63)	(*n* = 28)	(*n* = 150)	
Patient felt the trial offered the best available treatment	49 (78 %)	16 (57 %)	57 (39 %)	122 (51 %)
Patient felt the trial result could benefit others	53 (84 %)	24 (86 %)	143 (96 %)	220 (92 %)
Patient wanted to contribute to scientific research	37 (59 %)	16 (57 %)	111 (74 %)	164 (68 %)
Patient felt they would be monitored more closely	28 (44 %)	9 (32 %)	42 (28 %)	79 (33 %)
Patient felt they would have better quality care	20 (32 %)	5 (18 %)	24 (16 %)	49 (20 %)
Patient’s family were keen for patient to participate	24 (38 %)	8 (29 %)	20 (13 %)	52 (22 %)
Patient trusted the doctor treating them	38 (60 %)	10 (36 %)	73 (49 %)	121 (50 %)
Patient felt that otherwise their cancer will get worse	17 (27 %)	3 (11 %)	13 (9 %)	33 (14 %)
Other reason	4 (6 %)	0 (0 %)	8 (5 %)	12 (5 %)

Key: CTIMP = Clinical Trial of an Investigational Medicinal Product, pre-screening = molecular pre-screening to determine potential eligibility for a specific CTIMP study

### Patients who declined a clinical trial

Ten of the 25 patients who declined a clinical trial completed the questionnaire. There was no pattern to the types of trial declined and no demographic differences between the patients who consented and those who declined. Reasons for declining a trial included: unwillingness to take the study drug/placebo or have additional research procedures, decision not to have any further treatment, general anxiety about cancer diagnosis/treatment and a belief that the standard treatment was more effective. Nine of the 10 patients stated that they were glad to have been approached about participating in cancer research.

### Patients’ views on cancer research and biopsies

Two hundred and twenty-one patients (96 %) who completed Questionnaire A were happy to have been approached about participating in cancer research and 225 out of 228 patients (99 %) believed that cancer research would help doctors better understand and treat cancer.

Fifteen patients (7 %) agreed/strongly agreed, 43 patients (19 %) were neutral and 164 patients (74 %) disagreed/strongly disagreed with the statement: ‘I have concerns about the use and storage of blood and tissue samples for research’. Regarding genetic research, 173 patients (78 %) agreed/strongly agreed, 25 patients (11 %) were neutral and 25 patients (11 %) disagreed/strongly disagreed with the statement: ‘I would agree to donate tissue for genetic research even if I was not told my genetic results’.

In response to the question: ‘would you participate in a trial that required you to have a repeat biopsy?’ 78 patients (34 %) answered ‘yes’, 95 patients (41 %) answered ‘maybe’, 48 patients (21 %) answered ‘no’ and 10 patients (4 %) did not answer the question. The results of a univariate analysis of patient factors and their association with patients’ views of research biopsies is shown in Table 4.

**Table 4:** Univariate analysis of factors influencing patients’ views on whether they would participate in a trial involving a research biopsy

Patient characteristic	Yes	Odds ratio (95 % CI)	*p* value^a^
	*N* (%)		
	(*n* = 78)		
Age			
< 65 years	37 (57 %)	1.0	
≥ 65 years	41 (67 %)	1.55 (0.75–3.21)	0.236
Gender			
Male	59 (64 %)	1.0	
Female	19 (56 %)	0.71 (0.32–1.58)	0.398
Tumour type			
Colorectal	30 (60 %)	1.0	
Oesophagogastric	37 (73 %)	1.76 (0.76–4.06)	0.184
Performance status (PS)			
0	30 (67 %)	1.0	
1	36 (59 %)	0.72 (0.32–1.61)	0.423
2	7 (70 %)	1.17 (0.26-5.16)	0.839
Previously participated in a clinical trial			
Yes	13 (65 %)	1.0	
No	64 (61 %)	1.19 (0.44–3.23)	0.733
Number of previous lines of treatment			
0	24 (47 %)	1.0	
1	31 (67 %)	2.33 (1.02–5.31)	0.045
2+	22 (79 %)	4.13 (1.43–11.9)	0.009
Type of treatment			
Palliative	60 (69 %)	1.0	
Curative	18 (46 %)	0.39 (0.18–0.84)	0.015
CTIMP			
No	44 (60 %)	1.0	
Yes	19 (53 %)	0.74 (0.33–1.65)	0.457

^a^ *p* values compare the proportion of patients who answered ‘yes’: e.g. 67 % for PS0 versus 59 % for PS1 and 70 % for PS2

Key: CTIMP = Clinical Trial of an Investigational Medicinal Product, pre-screening = molecular pre-screening to determine potential eligibility for a specific CTIMP study

### Patients’ understanding of their cancer diagnosis

One hundred and eighty-three patients (79 %) correctly stated their diagnosis, 14 patients (6 %) wrote an incorrect answer (mainly indicating a metastatic site as their type of cancer) and 34 patients (15 %) did not answer the question. Patients under the age of 65 years were more likely to answer the question correctly (87 % versus 71 %, odds ratio 2.69, 95 % CI 1.38– 5.25, *p* = 0.004).

### Patients’ views on the written trial information

**Fig. 3:**
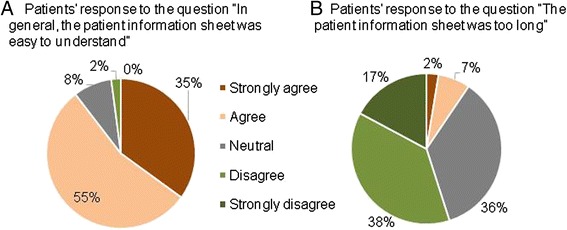
Patients’ views on the length and readability of the patient information sheet

**Fig. 4:**
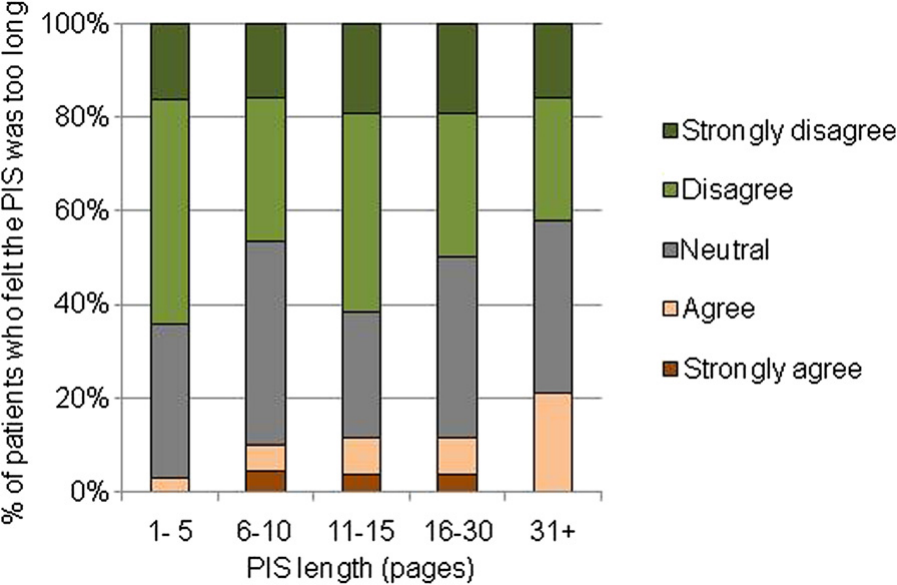
Relationship between patient information sheet (PIS) length and patients’ views on PIS length

The mean PIS length was 17 pages (median 4 pages, range 3–50 pages) and was influenced by trial type (CTIMP: 23 pages, non-CTIMP: 8 pages, pre-screening: 5 pages, *p* < 0.001). Two hundred and fifteen patients (90 %) felt the PIS was easy to understand and 22 patients (9 %) felt the PIS was too long (see Fig. 3). One patient admitted to not having read the information. There was a poor correlation between PIS length and patients’ views on whether the PIS was too long (see Fig. 4). All 10 patients who declined a study and completed the questionnaire believed the PIS was easy to understand and none of these patients felt the PIS was too long.

**Fig. 5:**
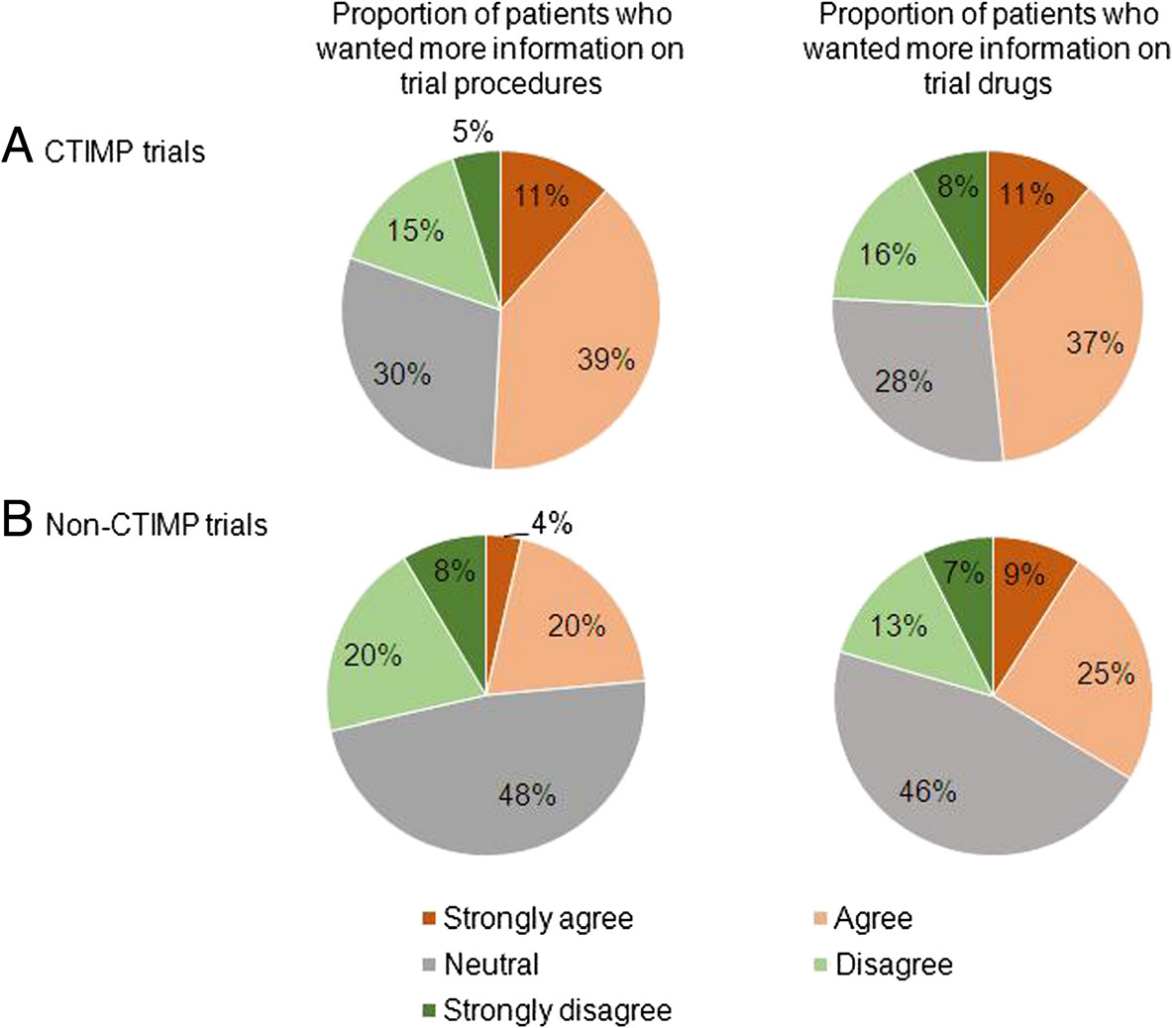
Patients’ views on the amount of information provided in the patient information sheet. Key: CTIMP = Clinical Trial of an Investigational Medicinal Product

Patients’ views on the content of the PIS are shown in Fig. 5. Thirty-six patients (15 %) looked up additional information about the trial (CTIMP: 32 %, pre-screening: 14 %, non-CTIMP: 7 %). Some patients wrote free-text comments requesting additional information: e.g. ‘more stats on the progress of the trial’ and ‘I found reading the actual trial protocol particularly useful’.

### Patients’ views on the verbal explanation, trial discussions and the consent process

The verbal explanation of the trial was rated as excellent, good, fair or poor by 100 (40 %), 124 (50 %), 14 (6 %) and 1 (0.4 %) patients respectively. All 10 patients who declined a study and completed the questionnaire rated the verbal explanation as excellent/good.

Two hundred and nineteen patients (88 %) discussed the trial with 1 or more people (CTIMP: 97 %, pre-screening: 96 %, non-CTIMP: 83 %), usually a family member (see Table 5). Two hundred and thirty-one patients (93 %) felt they had been given enough time to consider whether they wished to participate in the trial. Twenty-two pre-screening trial patients (79 %), 103 non-CTIMP trial patients (66 %) and 35 CTIMP trial patients (54 %) would have been willing to consent to the trial on the same day they received the PIS.

**Table 5:** People with whom patients discussed participation in a clinical trial

Person	*N* (%)
	(*n* = 249)
Spouse/partner	171 (68 %)
Son/daughter	64 (26 %)
Sibling	56 (23 %)
Friend	37 (15 %)
Parent	16 (6 %)
GP	13 (5 %)
Grandchild	7 (3 %)
Other^a^	11 (4 %)

^a^Other included: other family members, work colleague/boss, other doctors, Macmillan nurse, nutrition advisor/mind coach and their insurance company.

Many patients discussed trial participation with more than one person

### Feedback of study results

One hundred and seventy-three patients (75 %) felt patients should be told trial results, 13 patients (6 %) felt they should not be told and 40 patients (17 %) were unsure. A higher proportion of CTIMP trial patients felt participants should be told trial results (88 % versus 68 % for non-CTIMP trials, odds ratio 3.29, 95 % CI 1.46–7.45, *p* = 0.004). One hundred and ten patients (52 %) felt results should be provided by post, 91 patients (42 %) felt these should be discussed in clinic, 33 patients (16 %) thought via a website and 2 patients added free-text comments suggesting via Email.

## Discussion

The majority of patients in our survey were happy to be approached about participating in cancer research and were keen to participate in clinical trials. We chose to develop our own survey as existing surveys, such as the UK National Cancer Experience Survey, cover a broad range of topics and only include a few questions on clinical trials. Although our survey was less well-validated, it allowed us to determine the proportion of patients who consented to a clinical trial as well as investigate patients’ views on cancer research in more detail. Reassuringly, our results were consistent with the UK National Cancer Experience Survey, which reported that 95 % of patients who had research discussed with them were happy to have been asked and 53 % of patients with whom research was not discussed would have been happy to have been asked [17]. However, patients who have just started their first treatment for cancer are less likely to participate in cancer research and it appears that as time increases from diagnosis, patients are more positive about engaging with research [6, 18]. This may reflect the availability of clinical trials, but may also be influenced by factors such as the psychological impact of a recent cancer diagnosis. Patients were particularly willing to consent to non-CTIMP trials, possibly due to the less interventional nature of these studies. We had originally planned to compare the characteristics of the patients who consented to a trial with those who declined a trial, but this was not possible due to the small number of patients who declined a trial.

However, although a high proportion of patients consented to a trial, 36 % of patients subsequently failed screening for CTIMP/pre-screening trials. Screen failures may become increasingly problematic due to the growing number of biomarker selected/stratified trials. Twenty-eight percent of our trial portfolio involved tissue analysis prior to patient randomisation (either to determine eligibility or for stratification), resulting in 66 % of screen failures being caused by patients’ molecular profiles or a lack of tissue for analysis. This has important logistical and workload implications, as staff time is required for trial set-up, patient recruitment and specimen coordination, even though many patients are subsequently ineligible for the trial.

In agreement with other studies, we found that patients’ decisions to participate in research are influenced by multiple factors including altruism, trust in their treating physician and beliefs that they would receive superior treatment, closer monitoring and better quality care [11, 12, 19–23]. However, the ‘most important’ reason varied according to factors such as trial type and treatment intent. Although 52 % of patients consenting to CTIMP trials were motivated by a belief that the trial was the best treatment option available, 25 % of patients participated for altruistic reasons. Interestingly, 15 % of non-CTIMP trial respondents felt that the trial offered the best treatment available. For some trials (e.g. molecular profiling trials), this was a logical response. However, other trials had no direct patient benefit and, therefore, some patients seem to have misunderstood the trials’ aims. Other studies have shown that patients can misunderstand the potential personal benefit from clinical trials [13, 20] and it is important to ensure that patients clearly understand the purpose of any trial and any trial-related procedures.

Translational research is increasingly important and patients’ opinions of tissue and genetic research should be considered by researchers and ethics committees when assessing translational research protocols. Reassuringly, only 7 % of our patients had concerns about the use and storage of blood/tissue samples for research, and indeed, many patients strongly felt that this was not an issue. This is supported by the results of other studies, which demonstrate that the majority of cancer patients would allow the use of their tissue for research [13, 24, 25]. In our survey, 78 % of patients would agree to donate tissue for genetic research, even if they were not told their genetic results, indicating patient support for genomic research into cancer. However, these results should be interpreted with caution as no information was provided in our questionnaire regarding the potential implications of genetic results, so patients’ understanding and knowledge of any potential issues is unknown.

Biopsies are becoming an increasingly common component of clinical trials and 42 % of our trials involved an optional or mandatory biopsy. There have been ethical concerns about mandatory research biopsies: e.g. a lack of alternative treatment options and patients’ understanding of biopsy risks and the purpose of the biopsy [14, 26–28]. Although some patients would not participate in trials involving a biopsy, it is important to highlight that 75 % of our patients stated they would consider participating in a trial involving a repeat biopsy. This is significant as staff and ethics committee assumptions regarding patients’ attitudes to biopsies may not be accurate. For example, a survey of patients, medical oncologists and Institutional Review Board (IRB) members on the issue of mandatory research biopsies demonstrated that oncologists and IRB members may over-estimate the anxiety associated with biopsies and that patients would accept a higher risk of biopsy-related complications than oncologists/IRB members [13]. Furthermore, staff may be reluctant to discuss biopsies with older-aged patients or patients with a poorer PS due to assumptions that these patients would not wish to undergo any additional procedures. Interestingly, age and PS did not significantly impact on the willingness of our patients to consider a biopsy and we feel that these factors need not be a barrier to discussing biopsies with patients, allowing them to make their own decisions.

Patients being treated with palliative intent and patients who have received more lines of treatment appear to be more willing to consider a repeat biopsy. This could be due to a number of reasons, including willingness to participate in anything that could potentially benefit them, increasing altruistic motives or a better understanding of cancer and its treatment. Patients with a recent diagnosis of cancer are often keen to start treatment as soon as possible, and may be deterred by concerns regarding potential biopsy-related delays, may feel psychologically overwhelmed by their diagnosis and, as previously mentioned, are less likely to engage in research [18]. However, these results should be interpreted with caution due to the small number of patients in the subgroup analyses and because trials in more advanced lines of treatment were more likely to contain a biopsy component. Further research is needed into patients’ views on biopsies, particularly as it has been suggested that previous negative biopsy experiences may discourage patients from future biopsies [29].

Lengthy trial information can be off-putting to patients [22] and healthcare staff may feel that patients are overwhelmed by the amount of information provided, particularly as they have often also received information about their cancer and their standard treatment options. In addition, lengthy trial information can lead to a poor recall of risks and it has been suggested that shorter information leaflets might lead to better informed consent [30]. Indeed, one patient admitted to not having read the PIS, and this is not unique to our study [31]. PIS are often reviewed/tested by patients prior to submission to ethics committees, and this can result in amendments to their wording and layout that make the final version easier to understand [32]. However, even though the average PIS length was 17 pages, 55 % of patients clearly stated they did not feel it was too long and 48–50 % of patients approached about CTIMP studies would have liked more information on the study drugs/procedures. Therefore, there does seem to be a group of patients who wish to know more details about the trial, and indeed many of the free-text comments expressed a desire for more detailed information. The majority of patients discussed the trial with family members, and so patients’ relatives may also wish to have more detailed trial information.

However, approximately 20–28 % of our patients did not want more information on the trial drugs/procedures and other studies have shown that patients feel that the amount of information provided is sufficient [11]. A one-size-fits-all approach is, therefore, unlikely to suit the needs of every patient, and strategies to tailor information according to the individual patient’s wishes should be explored. Fifteen percent of patients looked up additional information (rising to 32 % for CTIMP studies) and, therefore, one strategy could be to provide a link in the PIS to a website containing further trial information. This would have the advantage of providing a resource for interested patients without overloading patients who are satisfied with the level of information already provided.

Patients who enrol quickly into clinical trials may not feel that they fully understand the implications of trial participation [33] and, therefore, ethics applications may state that patients will be given at least 24 hours to consider the information provided. However, some patients find it inconvenient to return specifically to sign consent and do not wish to delay screening procedures/treatment. This can be a particular issue for pre-screening trials, which involve biomarker testing of archival tissue to identify patients who might be eligible for corresponding CTIMP trials. As 79 % of patients approached about pre-screening trials would have been willing to consent on the same day as receiving the PIS, perhaps patients who wish to consent on the same day should be able to do so (with the important caveat of ensuring patients do not feel pressurised into signing consent).

Although 75 % of patients in our survey (rising to 89 % for CTIMP trials) felt participants should be informed of trial results, these are not always effectively communicated to patients. The question was specifically worded to highlight the fact that results may not be available for many years, although it did not directly state that patients may be deceased at the point when final study results are available. Our results are comparable to those from other studies [22, 34–36], indicating that the majority of patients feel they should be offered the trial results, not only for their personal interest and satisfaction, but also out of respect for their contribution to the trial [22]. One of the concerns regarding the feedback of trial results is the potential for psychological distress (e.g. due to randomisation to an arm with inferior outcomes) [35, 36]. However, although receiving results can be distressing [37, 38], this does not necessarily lead to regret at receiving results and some patients suggested that the satisfaction of knowing the results outweighed the potential distress of hearing bad news [36]. Indeed, one study demonstrated that 84 % of patients would like to receive results from a negative trial [36] and receiving study results may improve participants’ research experience [39]. Furthermore, not receiving results may deter patients from participating in future trials [34].

However, the optimal content, timing and method of providing results to participants is currently unclear, with our patients being divided as to whether results should be provided in clinic or via a letter. Some patients wish to know their personal results, others want the overall study results and many wish to know both [36]. In addition, patients are keen to receive regular updates on the trial’s progress [22, 36, 40]. Many sponsors provide participating sites with newsletters providing progress updates, so one strategy could be to provide a lay version of these newsletters to interested patients as well as a lay summary when the results are available.

When considering the applicability of our results to other patient populations, there are a number of factors that should be considered. Firstly, the structure of the UK health service (which is not dependent on patients’ health insurance) may facilitate trial participation [41]. Secondly, our patients were predominantly white, middle-class men with low levels of social deprivation. Although we did not specifically assess the educational level or health literacy of our patients, it is likely that our patients are more highly educated than patients from more deprived socioeconomic backgrounds, and issues such as lack of support, financial worries and difficulties with transport may be less problematic for our patients in comparison to patients from socially deprived areas [42]. Despite this, it is concerning that 21 % of patients either did not correctly state their diagnosis or left this question blank. It is uncertain whether the patients who did not answer the question accidentally missed the question or did not know what to write. If patients have not accurately understood their diagnosis, then they are less likely to fully understand the trial information. In addition, this survey only included patients with GI malignancies or lymphoma, and their views (particularly on biopsies) may differ from patients with other types of cancer. Also, the trial portfolio was heterogeneous in nature and the characteristics of the individual trials (e.g. palliative versus curative, study phase) and PIS may have influenced patients’ responses.

In addition, whether patients’ answers to the questionnaire truly reflect their views on the PIS is uncertain. Patients’ responses may be influenced by ‘social desirability’: i.e. they may give a socially desirable response if they know someone else will read their answers [43]. Furthermore, patients may falsely believe they have understood the PIS. For example, 93 % of patients in one phase I study stated they understood most or all of the information provided, but only 33 % were able to state the purpose of the trial in which they were participating [12]. Additionally, some of the questions were hypothetical in nature for some patients, as they were not relevant to the type of trial for which they had received a PIS and this may have influenced their responses.

However, it is important to highlight that although RM is a specialist cancer centre and patients referred from elsewhere in the UK for consideration of clinical trials may be more motivated to participate in cancer research, the majority of patients in our survey lived locally and were not referred to RM specifically for a trial. In addition, one of the main strengths of this survey is that a high proportion of patients who consented to a trial also completed the questionnaire (thereby minimising any potential bias between questionnaire responders and non-responders).

## Conclusions

In summary, this survey provides an insight into the views of patients on cancer research. The majority of patients were happy to have been approached about participating in clinical trials, and only a small proportion of patients declined a clinical trial. Although a major motivating factor was the possibility of improving their own treatment, many patients were also keen to help others and to contribute to scientific research. This extends to the use of tissue samples for research and to the consideration of research biopsies. We recommend that clinical trials and research biopsies are discussed with potentially eligible patients (including older-aged patients), to provide interested patients with the opportunity to participate in research. New strategies for tailoring the information needs to the individual patient, methods for disseminating trial results to participants and incorporating options for feedback of results into the initial trial consent process should be considered.

## Additional files

Additional file 1:
**Questionnaire A.** Description: questionnaire given to patients who consented to a clinical trial. (DOC 166 kb) Additional file 2:
**Questionnaire B.** Description: questionnaire given to patients who declined a clinical trial. (DOC 156 kb)

## Abbreviations

CTIMP: Clinical Trial of an Investigational Medicinal Product
GI: gastrointestinal
HER2: human epidermal growth factor receptor 2
IRB: Institutional Review Board
NIHR: National Institute for Health Research
NRS: National Readership Survey
Non-CTIMP: clinical trial not involving an investigational medicinal product
PIS: patient information sheet
PS: performance status
RM: Royal Marsden
UK: United Kingdom
