# Supplementary material for: Patients’ willingness to participate in clinical trials and their views on aspects of cancer research: results of a prospective patient survey
Source: Trials. 2016 Jan 9;17:17. doi: 10.1186/s13063-015-1105-3 (PMC4706669; doi:10.1186/s13063-015-1105-3)
Supplement: Additional file 1: — Questionnaire A. Description: questionnaire given to patients who consented to a clinical trial. (DOC 166 kb) [file 13063_2015_1105_MOESM1_ESM.doc]

Questionnaire A

Service evaluation no:

**
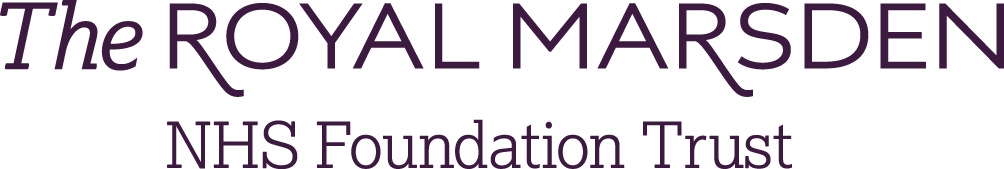
**

# SPECIFIC: Service evaluation of Patient Experience of Clinical trials and Factors Influencing Clinical trial entry

*We are asking patients who are being treated at The Royal Marsden to complete this questionnaire. It is designed to help us improve the experiences of patients with regards to clinical trials.* ***All responses provided will be kept strictly confidential and they will not influence or affect your treatment in any way****. Most questions should be answered by ticking one or more boxes (*🞏). *If you do not wish to answer a question then leave it blank. If you have any queries about this questionnaire, or any difficulties in completing it, then please do not hesitate to ask for assistance from your nurse or doctor.*

1. This questionnaire is about the_________________________________ clinical trial. Have you completed this questionnaire today about a different trial?

*Please tick one box* Yes 🞏 No 🞏

*If yes, please write the name of the trial below and then go to question 13.*

*________________________________________________________________*

*If no, please continue to question 2.*

1. Were you happy to be approached about participating in cancer research?

*Please tick one box* Yes 🞏 No 🞏

1. How many different trials were discussed with you within the last 2 weeks?

*Please tick one box* 1 🞏 2 🞏   3 🞏 4 🞏 More than 4 🞏

1. Have you previously participated in a clinical trial?

*Please tick one box* Yes 🞏 No 🞏

If yes, how many trials? *Please tick one box* 1 🞏 2 🞏  3 🞏 More than 3 🞏

1. Were you referred to The Royal Marsden specifically for a clinical trial?

*Please tick one box* Yes 🞏 No 🞏

1. How long have you been a patient at The Royal Marsden? *Please tick one box*

Less than 2 weeks 🞏 2-4 weeks 🞏 1-6 months 🞏 More than 6 months 🞏

1. What type of cancer do you have? *Please write your answer below*

*______________________________________________________________________*

1. How long does it usually take you to get to The Royal Marsden?

*Please tick the most appropriate*

Less than 30 minutes 🞏 30 minutes – 1 hour 🞏 1 hour – 1 ½ hours 🞏

1. ½ - 2 hours 🞏 More than 2 hours 🞏

1. How do you usually travel to The Royal Marsden? *Please tick the most appropriate*

By public transport 🞏 I drive myself 🞏 By bicycle 🞏

A friend or relative drives me 🞏   On foot 🞏       By taxi 🞏

By hospital transport 🞏

1. Please indicate how strongly you agree or disagree with each of the following statements. *Please tick one box per statement*

|  | **Strongly agree** | | **Agree** | | **Neutral** | | **Disagree** | | **Strongly disagree** | |
| --- | --- | --- | --- | --- | --- | --- | --- | --- | --- | --- |
| I believe cancer research will help doctors better understand and treat cancer | | 🞏 | | 🞏 | | 🞏 | | 🞏 | | 🞏 |
| I have concerns about the use and storage of blood and tissue samples for research | | 🞏 | | 🞏 | | 🞏 | | 🞏 | | 🞏 |
| I would agree to donate tissue for genetic research even if I was not told my genetic results | | 🞏 | | 🞏 | | 🞏 | | 🞏 | | 🞏 |

1. Would you participate in a trial that required you to have a repeat biopsy?

*Please tick one box* Yes 🞏 No 🞏 Maybe 🞏

1. Clinical trial results are often not available for many years. Do you think patients should be told the results of trials when they become available?

*Please tick one box* Yes 🞏 No 🞏 Maybe 🞏

If yes, how do you think this information should be provided?

*Please tick as many as appropriate* By post 🞏 On a website 🞏 In clinic 🞏

1. Did you discuss your participation in this trial with any of the following people?

*Please tick as many as appropriate*

My Spouse/Partner 🞏 My Mother/Father 🞏 My GP 🞏

My Brother(s)/Sister(s) 🞏 One or more friends 🞏

My son(s)/daughter(s) 🞏 My granddaughter(s)/grandson(s) 🞏

Somebody else *(please give details):* ________________________________________

1. Did you look up additional information regarding the trial?

*Please tick one box* Yes 🞏 No 🞏

1. How many times had you previously met the doctor who first told you about the trial?

*Please tick one box*

Never 🞏   Once 🞏  2-3 times 🞏 More than 3 times 🞏

1. Who gave you the most verbal information about the trial? *Please tick one box*

Consultant 🞏   Clinical Research Fellow 🞏 Registrar 🞏

Research nurse 🞏   Clinical nurse specialist 🞏 Don't know  🞏

Other *(please specify below)*

_________________________________________________

1. Please indicate how strongly you agree or disagree with each of the following statements about the patient information sheet. *Please tick one box per statement*

|  | **Strongly agree** | **Agree** | **Neutral** | **Disagree** | **Strongly disagree** | **Not applicable** |
| --- | --- | --- | --- | --- | --- | --- |
| In general, the patient information sheet was easy to understand | 🞏 | 🞏 | 🞏 | 🞏 | 🞏 | 🞏 |
| I would have liked the patient information sheet to tell me more about any additional research procedures | 🞏 | 🞏 | 🞏 | 🞏 | 🞏 | 🞏 |
| I would have liked the patient information sheet to tell me more about the drugs in the trial | 🞏 | 🞏 | 🞏 | 🞏 | 🞏 | 🞏 |
| The patient information sheet was too long | 🞏 | 🞏 | 🞏 | 🞏 | 🞏 | 🞏 |

1. Please rate the verbal explanation you received of the trial. *Please tick one box*

Excellent 🞏 Good 🞏 Fair 🞏 Poor 🞏

1. Why did you agree to participate in the trial?

*Please tick as many as appropriate*

1. I thought the trial offered the best treatment available 🞏
2. I believe results from the trial could benefit other patients in the future 🞏
3. I want to contribute to scientific research 🞏
4. I believe I will be monitored more closely as part of this trial 🞏
5. I believe the quality of care I receive will be better as part of this trial 🞏
6. My family were keen for me to participate 🞏
7. I trusted the doctor treating me 🞏
8. I think my cancer will get worse unless I take part in the trial 🞏
9. Other *(please give details below)*

________________________________________________________________

1. Which of the reasons above was the most important reason for you deciding to participate? *Please write the letter (from A-I) that corresponds to the most important reason in the box provided*
2. Did you feel you were given enough time to consider whether or not you wished to participate in this trial? *Please tick one box* Yes 🞏 No 🞏
3. Did you feel under pressure to participate in the trial?

*Please tick one box* Yes 🞏 No 🞏

If yes, who by? *Please tick as many as appropriate*

A Royal Marsden doctor 🞏 The research nurse 🞏 A family member 🞏 

Other (please specify) ___________________________________________________

1. Would you have been happy to consent to this trial on the same day that you were given the patient information sheet? *Please tick one box* Yes 🞏 No 🞏
2. Is there anything that you feel we could improve upon? *Please write your answer below*
3. Any other comments? *Please write your answer below*

**Thank you for completing this questionnaire**

We would also like to find out more about patients’ experiences of participating in clinical trials at The Royal Marsden. This will help us improve the service for future patients and help us to develop new clinical trials. This would involve patients completing another questionnaire in clinic.

If you would be interested in participating in any future surveys about clinical trials please tick the box, write your name and date of birth below and sign your agreement. You are under no obligation to complete this section.

I agree to be contacted in the future regarding my experiences of clinical trials 🞏

Name: ________________________________________________________________

Date of birth: ________________________

Signature: _______________________________________ Date: _________________

For office use only

Hospital number:
