# Supplementary material for: Patients’ willingness to participate in clinical trials and their views on aspects of cancer research: results of a prospective patient survey
Source: Trials. 2016 Jan 9;17:17. doi: 10.1186/s13063-015-1105-3 (PMC4706669; doi:10.1186/s13063-015-1105-3)
Supplement: Additional file 2: — Questionnaire B. Description: questionnaire given to patients who declined a clinical trial. (DOC 156 kb) [file 13063_2015_1105_MOESM2_ESM.doc]

Questionnaire B

Service evaluation no:


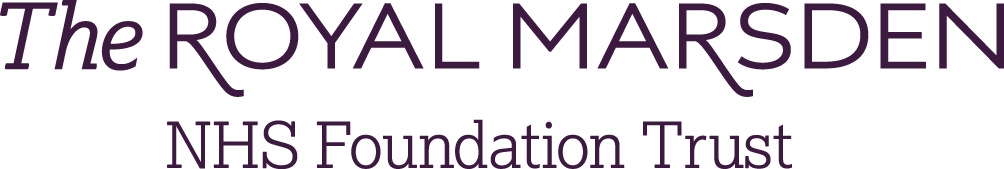


# SPECIFIC: Service evaluation of Patient Experience of Clinical trials and Factors Influencing Clinical trial entry

*We are asking patients who are being treated at The Royal Marsden to complete this questionnaire. It is designed to help us improve the experiences of patients with regards to clinical trials.* ***All responses provided will be kept strictly confidential and they will not influence or affect your treatment in any way****. Most questions should be answered by ticking one or more boxes (*🞏). *If you do not wish to answer a question then leave it blank. If you have any queries about this questionnaire, or any difficulties in completing it, then please do not hesitate to ask for assistance from your nurse or doctor.*

1. This questionnaire is about the_________________________________ clinical trial. Have you completed this questionnaire today about another trial?

*Please tick one box* Yes 🞏 No 🞏

*If yes, please write the name of the trial(s) below and then go to question 11.*

*____________________________________________________________________*

*If no, please continue to question 2.*

1. Were you happy to be approached about participating in cancer research?

*Please tick one box* Yes 🞏 No 🞏

1. How many different trials were discussed with you within the last 2 weeks?

*Please tick one box* 1 🞏    2 🞏   3 🞏   4 🞏 More than 4 🞏

1. Have you previously participated in a clinical trial?

*Please tick one box* Yes 🞏 No 🞏

If yes, how many trials? *Please tick one box* 1 🞏 2 🞏  3 🞏 More than 3 🞏

1. Were you referred to The Royal Marsden specifically for a clinical trial?

*Please tick one box* Yes 🞏 No 🞏

1. How long have you been a patient at The Royal Marsden? *Please tick one box*

Less than 2 weeks 🞏 2-4 weeks 🞏 1-6 months 🞏 More than 6 months 🞏

1. What type of cancer do you have? *Please write your answer below*

*______________________________________________________________________*

1. How long does it usually take you to get to The Royal Marsden?

*Please tick the most appropriate*

Less than 30 minutes 🞏 30 minutes – 1 hour 🞏 1 hour – 1 ½ hours 🞏

1 ½ - 2 hours 🞏 More than 2 hours 🞏

1. How do you usually travel to The Royal Marsden? *Please tick the most appropriate*

By public transport 🞏 I drive myself 🞏 By bicycle 🞏

A friend or relative drives me 🞏   On foot 🞏       By taxi 🞏

By hospital transport 🞏

1. Please indicate how strongly you agree or disagree with each of the following statements. *Please tick one box per statement*

|  | **Strongly agree** | | **Agree** | | **Neutral** | | **Disagree** | | **Strongly disagree** | |
| --- | --- | --- | --- | --- | --- | --- | --- | --- | --- | --- |
| I believe cancer research will help doctors better understand and treat cancer | | 🞏 | | 🞏 | | 🞏 | | 🞏 | | 🞏 |
| I have concerns about the use and storage of blood and tissue samples for research | | 🞏 | | 🞏 | | 🞏 | | 🞏 | | 🞏 |
| I would agree to donate tissue for genetic research even if I was not told my genetic results | | 🞏 | | 🞏 | | 🞏 | | 🞏 | | 🞏 |

1. Did you discuss your participation in this trial with any of the following people?

*Please tick as many as appropriate*

My Spouse/Partner 🞏 My Mother/Father 🞏 My GP 🞏

My Brother(s)/Sister(s) 🞏 One or more friends 🞏

My son(s)/daughter(s) 🞏 My granddaughter(s)/grandson(s) 🞏

Somebody else *(please give details):* ________________________________________

1. Did you look up additional information regarding the trial?

*Please tick one box* Yes 🞏 No 🞏

1. How many times had you previously met the doctor who first told you about the trial?

*Please tick one box*

Never 🞏   Once 🞏  2-3 times 🞏 More than 3 times 🞏

1. Who gave you the most verbal information about this clinical trial? *Please tick one box*

Consultant 🞏   Clinical Research Fellow 🞏 Registrar 🞏

Research nurse 🞏   Clinical nurse specialist 🞏    

Other *(please specify below)*

_________________________________________________

1. Please indicate how strongly you agree or disagree with each of the following statements about the patient information sheet. *Please tick one box per statement*

|  | **Strongly agree** | **Agree** | **Neutral** | **Disagree** | **Strongly disagree** | **Not applicable** |
| --- | --- | --- | --- | --- | --- | --- |
| In general, the patient information sheet was easy to understand | 🞏 | 🞏 | 🞏 | 🞏 | 🞏 | 🞏 |
| I would have liked the patient information sheet to tell me more about any additional research procedures | 🞏 | 🞏 | 🞏 | 🞏 | 🞏 | 🞏 |
| I would have liked the patient information sheet to tell me more about the drugs in the trial | 🞏 | 🞏 | 🞏 | 🞏 | 🞏 | 🞏 |
| The patient information sheet was too long | 🞏 | 🞏 | 🞏 | 🞏 | 🞏 | 🞏 |

1. Please rate the verbal explanation you received of the trial. *Please tick one box*

Excellent 🞏 Good 🞏 Fair 🞏 Poor 🞏

1. Did you feel you were given enough time to consider whether or not you wished to participate in this trial? *Please tick one box* Yes 🞏 No 🞏
2. Did you feel under pressure to participate in this trial?

*Please tick one box* Yes 🞏 No 🞏

If yes, who by? *Please tick as many as appropriate*

A Royal Marsden doctor 🞏 The research nurse 🞏 A family member 🞏 

Other (please specify) ___________________________________________________

1. Why did you decide not to participate in this trial?

*Please write your answer in the box below and underline the most important reason*

1. Is there anything that you feel we could improve upon? *Please write your answer below*
2. Any other comments? *Please write your answer below*

**Thank you for completing this questionnaire**
